# Renal Tubular Acidosis Leading to Severe Hypokalemia: An Uncommon Adverse Effect of Nonsteroidal Anti-inflammatory Drugs

**DOI:** 10.7759/cureus.103872

**Published:** 2026-02-18

**Authors:** Abdullah Al Kahil

**Affiliations:** 1 Internal Medicine, University Hospitals, Parma, USA

**Keywords:** hypokalemia, ibuprofen, metabolic acidosis, nephrotoxicity, nsaids, renal tubular acidosis

## Abstract

Nonsteroidal anti-inflammatory drugs (NSAIDs) are commonly used over-the-counter pain-relieving medications. They are not free from side effects, including effects on the kidneys. We present the case of an elderly woman who presented with severe fatigue and generalized weakness. Her medical history was significant for osteoarthritis, for which her pain was controlled with ibuprofen and naproxen. She was found to have renal tubular acidosis (RTA), causing severe, persistent hypokalemia. The underlying etiology was concluded to be secondary to NSAID use, making this case unique. She gradually improved with electrolyte replacement and discontinuation of NSAIDs. This case emphasizes the importance of considering RTA in the differential diagnosis in the setting of unexplained hypokalemia and metabolic acidosis, especially in the absence of gastrointestinal losses or diuretic use. Discontinuation of NSAIDs with correction of electrolytes is crucial for recovery.

## Introduction

Nonsteroidal anti-inflammatory drugs (NSAIDs) have well-recognized renal side effects. Their effects on the kidneys can be seen in different aspects, including decreased renal perfusion, inhibition of prostaglandin actions on the renin-angiotensin system, and sodium and water retention [1,2]. Renal tubular acidosis (RTA) is a kidney disorder that causes a defect in kidney acid-base regulation, resulting in non-anion gap metabolic acidosis. This disorder has four different types with different features. In general, RTA is a rare complication with an unclear mechanism associated with NSAID use, most commonly, ibuprofen [3,4]. We present the case of an elderly woman who developed RTA due to chronic NSAID use, complicated by severe hypokalemia.

## Case presentation

A 71-year-old African American woman with a medical history of hypertension (managed with amlodipine 5 mg daily) and osteoarthritis of the left wrist and right knee, for which she chronically used ibuprofen 400 mg at night on an as-needed basis, with a recent increase in nighttime frequency, and naproxen 500 mg every 12 hours as needed during the daytime for pain control presented to the emergency department (ED) with four days of fatigue and generalized weakness. She reported being unable to get out of bed or care for herself. The patient denied vomiting, diarrhea, polyuria, use of herbal supplements, or recent alcohol consumption. She was brought to the ED by a family member who noticed worsening in her condition. On presentation, her vital signs were within normal limits. Physical examination revealed generalized weakness in all four extremities with muscle strength of 4/5. No other abnormal findings were noted.

Initial laboratory results (Table 1) demonstrated severe hypokalemia (1.9 mmol/L), bicarbonate of 18 mmol/L, and an initial anion gap of 20 mmol/L; repeat testing later that day showed an anion gap of 15 with a serum pH of 7.31. Additional findings included magnesium 1.48 mg/dL, albumin 4.3 g/dL, and an elevated beta-hydroxybutyrate level of 3.41 mmol/L. Urine studies revealed a potassium-to-creatinine ratio of 15 mmol/g, a urine anion gap of 10, and a urine pH of 6.5. Urine microscopy was unremarkable, and urine drug screen was positive for marijuana. Electrocardiography revealed a prolonged QT interval.

**Table 1:** Serum laboratory results

Test Name	Result	Reference Range
Glucose	117 mg/dL	70–99 mg/dL
Sodium	144 mmol/L, 143 mmol/L (repeated)	135–145 mmol/L
Potassium	1.9 mmol/L	3.5–5.1 mmol/L
Chloride	108 mmol/L, 108 mmol/L (repeated)	98–107 mmol/L
Bicarbonate	18 mmol/L, 20 mmol/L (repeated)	22–29 mmol/L
Anion Gap	20 mmol/L, 15 mmol/L (repeated)	8–16 mmol/L
Delta–Delta Gap	1.0, 0.8 (repeated)	-
Lactic Acid	2.4 mmol/L, 1.5 mmol/L (repeated)	0.5–2.2 mmol/L
Blood Urea Nitrogen	18 mg/dL	7–20 mg/dL
Creatinine	1.20 mg/dL	0.6–1.3 mg/dL
Calcium	9.7 mg/dL	8.6–10.2 mg/dL
Albumin	4.3 g/dL	3.5–5.0 g/dL
Creatine Kinase	755 U/L	30–200 U/L
Magnesium	1.48 mg/dL	1.7–2.2 mg/dL
Beta-Hydroxybutyrate	3.41 mmol/L	0.02–0.27 mmol/L

The patient was started on intravenous potassium chloride, magnesium sulfate, and aggressive intravenous hydration and was transferred to the intensive care unit. By the following day, serum potassium increased to 2.0 mmol/L and magnesium to 2.5 mg/dL, warranting continuation of intravenous potassium replacement. On hospital Day 3, the serum potassium remained low at 2.1 mmol/L. Repeat urine studies (Table 2) showed a urine potassium-to-creatinine ratio of 33 mmol/g, a urine anion gap of 25, and a urine pH of 6.5. These laboratory findings are consistent with underlying distal renal tubular acidosis causing renal potassium loss. Intravenous potassium supplementation was continued until later on Day 4, when serum potassium levels normalized. During the patient’s hospital stay, alternative causes were investigated.

**Table 2:** Urine laboratory results HPF: high-power field

Test Name	Result (Day 1)	Result (Day 3)
Urine Sodium (Random)	47 mmol/L	20 mmol/L
Urine Potassium (Random)	23 mmol/L	33 mmol/L
Potassium/Creatinine Ratio	15 mmol/g	33 mmol/g
Urine Chloride (Random)	60 mmol/L	30 mmol/L
Urine Creatinine (Random)	153.8 mg/dL	101.1 mg/dL
Urine Anion Gap	10 mmol/L	23 mmol/L
Urine pH	6.5	6.5
Urine Protein	(+1) on Urine Dipstick	(+1) on Urine Dipstick
WBC, Urine (/HPF)	1–5/HPF	-
RBC, Urine (/HPF)	3–5/HPF	-
Urine Casts and Crystals	None	-

After ruling out other potential etiologies and based on the patient’s clinical history, a diagnosis of NSAID-induced distal RTA was concluded. After the patient’s recovery, she was instructed to discontinue ibuprofen and naproxen at discharge, and an alternative pain management regimen was discussed.

## Discussion

This was an uncommon presentation of distal RTA complicated by severe hypokalemia caused by ibuprofen and naproxen use. Although the initial laboratory findings suggested high anion gap metabolic acidosis with starvation ketosis, repeated laboratory studies established the underlying diagnosis, supported by the onset of normal anion gap metabolic acidosis, inappropriately high urine pH, a positive urine anion gap in the absence of gastrointestinal potassium losses or diuretic use, and the absence of significant urine microscopy findings. The patient’s chronic NSAID use and clinical improvement after discontinuation strongly support our diagnosis.

The exact mechanism of NSAID-induced distal RTA is not fully understood. The assumed mechanisms of distal RTA (type 1) include inhibition of renal carbonic anhydrase II and suppression of distal tubular H⁺-ATPase activity [3,4]. This defect in distal H⁺/K⁺ exchange causes metabolic acidosis and renal potassium wasting, resulting in hypokalemia, which was seen in our case [5]. The presence of hypokalemia is contrary to the commonly observed potassium abnormality associated with NSAID use, as NSAIDs suppress prostaglandin synthesis and the renin-angiotensin-aldosterone system, usually resulting in hyperkalemia. This mechanism is also an important part of the pathophysiology of NSAID-induced type 4 renal tubular acidosis [6]. NSAIDs can induce other types of RTA through inhibiting carbonic anhydrase II, which is found in the proximal tubules, causing proximal RTA (type 2) or mixed RTA (type 3), which involves both proximal and distal RTA [7], as seen in the case reported by Dang MH et al. [8].

NSAIDs can affect renal function by inhibiting prostaglandin synthesis, resulting in afferent arteriolar vasoconstriction and reduced renal blood flow [1,2]. In our patient, this mechanism likely explained the transient pre-renal acute kidney injury (AKI), which resolved rapidly after intravenous fluid administration and discontinuation of NSAIDs; therefore, no further investigation was required. However, the continued presence of metabolic acidosis and severe hypokalemia despite recovery of renal function, together with a positive urinary anion gap and an inappropriately elevated urinary pH of 6.5 during metabolic acidosis, pointed to an additional functional tubular abnormality consistent with distal renal tubular acidosis. The pronounced tubular dysfunction observed in this patient may reflect individual susceptibility, potentially related to high-dose or prolonged NSAID exposure, advanced age, or underlying comorbidities, although definitive predictors remain poorly defined. Similar risk factors have been reported in the literature on NSAID-induced nephrotoxicity [9]. 

NSAIDs can also cause tubular dysfunction through structural injury by impairing kidney hemodynamics, leading to acute tubular necrosis or by direct tubulointerstitial inflammation, as seen in acute interstitial nephritis. Acute interstitial nephritis is often accompanied by extrarenal features such as fever, skin rash, arthralgias, and peripheral eosinophilia, none of which were present in our patient. Although these structural tubular injuries can sometimes be difficult to definitively exclude without renal biopsy, a biopsy was not indicated in this case, given the clinical course and rapid improvement in kidney function [9,10]. 

Resolution of distal RTA and normalization of serum potassium following discontinuation of NSAIDs, with potassium replacement, have been reported in similar cases in the literature, supporting the reversibility of this condition. This was seen in the case report by Ghimire A et al., who described a 66-year-old male with chronic ibuprofen use presenting with hypokalemia, non-anion gap metabolic acidosis, and AKI that resolved within 48 hours [11]. Thammineni et al. reported two patients with heavy over-the-counter ibuprofen use who developed life-threatening hypokalemia secondary to distal RTA, both requiring aggressive potassium replacement and improving after discontinuing ibuprofen [12].

## Conclusions

Nonsteroidal anti-inflammatory drugs-induced RTA, specifically types 1, can cause life-threatening hypokalemia and metabolic acidosis, which are uncommon adverse effects. Elderly patients and those with chronic NSAID use are at increased risk. Unexplained hypokalemia and metabolic acidosis, particularly in the absence of gastrointestinal losses or diuretic use, should raise suspicion for RTA. Careful review of the patient’s medication history, prompt discontinuation of NSAIDs, and aggressive correction of electrolyte abnormalities are crucial for recovery.
